# Error minimized LO modeling of electric vehicle integrated off-grid microgrids using Taylor-Laurent series expansion and BBO based optimization under stability and steady state constraints

**DOI:** 10.1038/s41598-026-43306-0

**Published:** 2026-03-15

**Authors:** Richa Chaudhary, V. P. Singh, Akhilesh Mathur, Mahipal Bukya

**Affiliations:** 1 Department of Electrical Engineering, Malaviya National Institute of Technology, Jaipur, 302017 Rajasthan India; 2 Department of Electrical Engineering, Poornima College of Engineering, Jaipur, 302022 Rajasthan India; 3 Department of Electrical and Electronics Engineering, Manipal Institute of Technology Bengaluru, Manipal Academy of Higher Education, Manipal, India

**Keywords:** Electric vehicle, Off-grid microgrid, Taylor series, Laurent series, Brown bear optimization algorithm, Energy science and technology, Engineering, Mathematics and computing

## Abstract

This paper presents an effective approach for lower-order (LO) modeling of an electric vehicle–integrated off-grid microgrid (OMG) system. The seventh-order system (SOS) of the OMG is reduced to a second-order model (SOM) while preserving the original system’s dynamic characteristics and ensuring computational efficiency. The Taylor series (TS) and Laurent series (LS) expansions are employed to simplify the complex system that plays a significant role in the reduction process. The expansion parameters of the higher-order system (HOS) of OMG and its lower-order model (LOM) are exploited to construct the fitness function. The proposed approach constructs three sub-objective functions based on TS and LS. These sub-objective functions are then combined into a single fitness function to obtain an improved LOM by enhancing the transient and steady-state responses with respect to the HOS of OMG. To minimize the error, the resultant fitness function is optimized using the brown bear optimization (BBO) algorithm. The optimization is performed under two key constraints: (i) ensuring zero steady-state error, and (ii) satisfying the Hurwitz stability criterion. To demonstrate the efficacy of the proposed LOM, it is compared with other LOMs obtained from different approximation techniques. The proposed LOM and other LOMs are graphically validated through step, impulse, Bode, Nichols, and Nyquist response comparisons with the HOS. Additionally, the performance error criteria (PEC), time-domain specifications (TDSs) and frequency domain specifications (FDSs) of the proposed LOM are compared with other LOMs using the HOS to establish the validation and applicability of the proposed method.

## Introduction

The rapid advancement of transportation, infrastructure, and industrial technologies has substantially increased global dependence on fossil fuels and large-scale electricity consumption, which results in harmful gas emissions and threatens environmental sustainability. To address these challenges, the adoption of electric vehicles (EVs) and renewable energy sources (RESs) has emerged as a promising solution to reduce reliance on petroleum-based fuels and conventional fossil fuel–based electricity^1^. The coordinated integration of EVs and renewable energy effectively reduces emissions of sulfur dioxide, nitrogen oxides, and greenhouse gases, thereby mitigating global warming and air pollution. The convergence of the electricity and transportation sectors not only supports the transition toward a low-carbon economy but also promotes long-term environmental preservation. However, the large-scale penetration of renewable energy and EV charging loads introduces voltage and frequency instability in power systems. To overcome this issue, the microgrid concept has gained significant attention in recent years^2,3^.

A microgrid is a network comprising distributed energy resources (DERs), renewable generation units, and EVs within a localized network interconnected at a common coupling point. It can operate either in grid-connected or stand-alone (off-grid) mode. The microgrid has its capability to function autonomously during grid outages or in regions without connected to utility grid. These autonomous capabilities are particularly advantageous in remote or rural areas where grid access is unavailable or the supplied electricity is unreliable. Microgrids improve the system efficiency by incorporating several DERs such as fuel cells, EVs, energy storage systems, microturbines, biodiesel engine generators, biogas turbine generators, photovoltaic systems, and wind turbine systems^4^. Furthermore, the integration of EVs into off-grid microgrids introduces an additional layer of flexibility and energy management capability. EVs can operate as mobile energy storage units, supporting load balancing, frequency regulation, and voltage stabilization through vehicle-to-grid and vehicle-to-home interactions. Their involvement increases the reliability and operational flexibility of OMG^5^.

The integration of several components in a microgrid poses a challenge as it forms a complex higher-order transfer function system. The higher-order systems (HOSs) have several disadvantages, like difficulties in the controller implementation, increases computational burden, longer simulation time, stability concerns, difficulties with real-time implementation, and issues in control system design. Due to this, the order reduction of an HOS is preferable^6^. The model order diminution (MOD) techniques are proposed to obtain a lower-order model (LOM). The benefits of MOD are that it decreases simulation time, makes controller design easier, easier model analysis, and improves computational efficiency in matrix inversion^7,8^.

In literature, several MOD methods are proposed for approximating HOSs. This paper^9^, author proposes to utilize the rank exponent-based lower-order (LO) modeling of a larger-order electric vehicle system of a fifth-order transfer function, and exploit the error minimization-based approach by utilizing the grey-wolf optimization algorithm. In^10^, a new gaussianly distributed spectral zero projection technique is developed for MOD for a large-scale system. In^11^, authors present a deep learning-based MOD technique for distributed parameter systems. In this paper^12^, author proposes a LO aggregate model based on balanced truncation technique to provide the preprocessing approach for real-time simulation of large-scale converters with inhomogeneous initial conditions in DC microgrid. In^13^, the author proposed an LOM of $15^{th}$ order of an interconnected wind-turbine system for single-area, two-area, and three-area interconnected systems with varying system order. Wang et al.^14^ approximated a heterogeneous integrated energy (HIE) system of orders twenty-seven and twenty to a sixth-order HIE model. Meanwhile, in^15^, authors investigated the reduction of continuous and discrete-time systems using the dominant pole retention technique. Furthermore^16^, also performed model reduction for a discrete interval system to demonstrate the effectiveness of MOD techniques. A survey of MOD techniques for physics-based lithium-ion battery management is explained in^17^. In^18^, an IRM-guided optimization framework was introduced, where the balanced residualization method generates an interim lower model to define a constrained search space, and the geometric mean optimization algorithm tunes the reduced model coefficients, resulting in improved stability and accuracy compared to conventional MOD approaches. In^19^, the authors propose the design and analysis of an innovative solar-powered EV charging system utilizing a TOSSI based on a character of triangular function. In paper^20^, the authors investigate load frequency control in a multi-area power system with diverse generation sources under normal and uncertain operating conditions. This work^21^ addresses the role of EVs as a distributed energy storage resource for enhancing power grid stability through smart charging and grid-support services.

This paper focuses on developing an effective LOM for HOS of OMG. In this work, the seventh-order system (SOS) of OMG is approximated into a second-order model (SOM). The core of proposed approach lies in constructing a three sub-objective function based on the Taylor series (TS) and Laurent series (LS) expansions of both the original HOS and the desired LOM. The three sub-objective functions are combined to form the resultant fitness function, which is then minimized using the brown bear optimization (BBO) algorithm, selected for its simplicity and effectiveness. The key constraints, such as steady-state accuracy and model stability, are imposed to ensure the reliability of the resulting LOM. The performance of the proposed technique is validated by comparing with other LOMs obtained from various MOD techniques. The graphical responses are presented through simulations involving step, impulse, Bode, Nichols, and Nyquist responses, along with normalized time domain specifications (TDSs) and error plots. Furthermore, a comparative analysis using TDSs and various performance error criteria (PEC) demonstrates that the proposed approach yields accurate and robust LOM when compared to other existing LOMs. The prime contribution of the presented article is as follows:

**Fig. 1:**
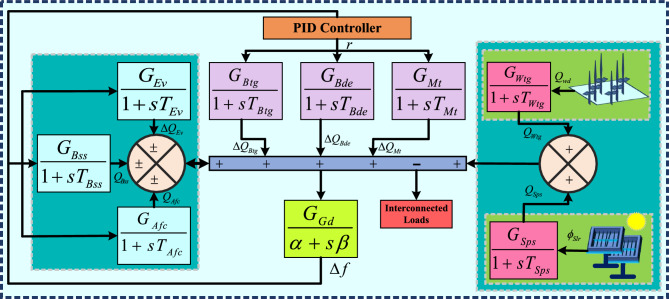
Transfer function model of an off-grid microgrid.

- A reduction of a SOS to a SOM is performed by an error minimization process employing TS and LS to both HOS and LOM of OMG.
- TS and LS are used to construct the fitness function to improve the transient response and steady state response of LOM.
- The fitness function is minimized using BBO algorithm. The minimization is subjected to two constraints, i.e., ensuring Hurwitz stability criterion and confirming steady state matching of HOS and LOM of OMG.
- For the validation of proposed approach, the responses are provided for the comparison assessment.

The structure of this paper is organized as follows: Section "Mathematical modeling of off-grid microgrid", outlines the mathematical modeling of OMG system and presents the problem formulation. In Section "Brown Bear Optimization (BBO) algorithm", the explanation of BBO algorithm is provided. Section "Results and discussion", presents the seventh-order OMG system for deriving a second-order OMG model, along with simulation results and comparative analysis. Finally, Section “Conclusion” concludes the study and highlights the future scope of this research.

## Mathematical modeling of off-grid microgrid

The generating components of OMGs is presented in Fig. 1. The OMG involves renewable source-based components, including solar photovoltaic systems (Sps), wind turbine generators (Wtg), biodiesel engine generators (Bde), biogas turbine generators (Btg), and micro turbines (Mt)-renewable only if operated on renewable fuels, and energy storage-based components such as electric vehicles (Ev), battery energy storage systems (Bss), and aqua electrolyser fuel cell systems (Afc). The mathematical representations of system components with their first-order transfer functions, gains, and time constants are presented in Table 1^6^.

**Table 1:** Mathematical representations of different components.

**Component**	**Transfer function**	**Gains**	**Time constants**	**Comments**
Ev	$Tf_{Ev}(s)=\dfrac{G_{Ev}}{1+sT_{Ev}}$	$G_{Ev}=1$	$T_{Ev}=0.1$	Converts stored electrical energy into wheel torque for commercial transportation.
Bss	$Tf_{Bss}(s)=\dfrac{G_{Bss}}{1+sT_{Bss}}$	$G_{Bss}=1$	$T_{Bss}=0.1$	Stores and releases energy to support demand peaks or maintain grid stability.
Afc	$Tf_{Afc}(s)=\dfrac{G_{Afc}}{1+sT_{Afc}}$	$G_{Afc}=0.002$	$T_{Afc}=0.5$	Electrolyzer produces hydrogen, and a fuel cell converts stored hydrogen into electricity.
Btg	$Tf_{Btg}(s)=\dfrac{G_{Btg}}{1+sT_{Btg}}$	$G_{Btg}=1$	$T_{Btg}=0.55$	Biogas from waste decomposition powers a turbine generator to produce electricity.
Bde	$Tf_{Bde}(s)=\dfrac{G_{Bde}}{1+sT_{Bde}}$	$G_{Bde}=1$	$T_{Bde}=0.148$	Biodiesel extracted from plants is used as a fuel for generating electrical power.
Mt	$Tf_{Mt}(s)=\dfrac{G_{Mt}}{1+sT_{Mt}}$	$G_{Mt}=1$	$T_{Mt}=1.5$	A small gas turbine generator that converts gaseous or liquid fuels into electricity and heat efficiently.
Wtg	$Tf_{Wtg}(s)=\dfrac{G_{Wtg}}{1+sT_{Wtg}}$	$G_{Wtg}=1$	$T_{Wtg}=1.5$	Converts wind energy into blade rotation, which is transformed into electrical energy.
Sps	$Tf_{Sps}(s)=\dfrac{G_{Sps}}{1+sT_{Sps}}$	$G_{Sps}=1$	$T_{Sps}=1.8$	Photovoltaic system that directly converts sunlight into electrical energy.
Gd	$Tf_{Gd}(s)=\dfrac{G_{Gd}}{\alpha +s\beta }$	$G_{Gd}=1$	$\alpha =0.012$, $\beta =0.2$	Gd represents the system behavior and response of a generation unit.

From Fig. 1 and tabular data provided in Table 1^22,23^, the overall output power is calculated by combining power generated by all the components, and is mathematically expressed in (1).

1 $$\begin{aligned} \Delta Q_{tot} = {\Delta Q_{Wtg}}+ {\Delta Q_{Sps}}+ {\Delta Q_{Mt}}+ {\Delta Q_{Bde}}+ {\Delta Q_{Btg}}\pm \Delta Q_{Ev} \pm \Delta Q_{Bss}\pm Q_{Afc} \end{aligned}$$

where $\Delta Q_{tot}$ is total generated power and $\Delta Q_{Wtg}$, $\Delta Q_{Sps}$, $\Delta Q_{Mt}$, $\Delta Q_{Bde}$, $\Delta Q_{Btg}$, $\Delta Q_{Ev}$, $\Delta Q_{Bss}$, and $\Delta Q_{Afc}$ are the variations of generated power by Wtg, Sps, Mt, Bde, Btg, Ev, Bss, and Afc, respectively. The mathematical expression for calculating the change in effective power ($\Delta Q_{eff}$) is given as,

2 $$\begin{aligned} \Delta Q_{eff}=\Delta Q_{tot}- \Delta Q_{il} \end{aligned}$$

where $\Delta Q_{il}$ is the consumed power by interconnected load. The mentioned components in Fig. 1 generate varying power, which gives rise to frequency deviations ${(\Delta f)}$ and is formulated in (3).

3 $$\begin{aligned} \Delta f=\frac{G_{Gd}}{\alpha +s\beta } \ \Delta Q_{eff} \end{aligned}$$

where, $\frac{G_{Gd}}{\alpha +s\beta }$ is generator dynamics. Considering ${(\Delta f)}$ as output and ${\Delta Q_{eff}}$ as input, the resulting transfer function is given as follows:

4 $$\begin{aligned} Tf_a{(s)}= \frac{\Delta f}{\Delta Q_{eff}} \end{aligned}$$

The generalized transfer function system of OMG is presented in (5).

### Representation of HOS and LOM

Suppose the transfer function of higher order of OMG can be expressed as,

5 $$\begin{aligned} {Tf}_{a}(s)= \frac{{\hat{\chi }_i(s)}}{{\hat{\kappa }_i(s)}} = \frac{{\hat{\chi }}_{0}+{\hat{\chi }}_{1}s+{\hat{\chi }}_{2}s^{2}+\ldots +{\hat{\chi }}_{a-1}s^{a-1}}{{\hat{\kappa }}_{0}+{\hat{\kappa }}_{1}s+{\hat{\kappa }}_{2}s^{2}+\ldots +{\hat{\kappa }}_{a}s^{a}} \end{aligned}$$

where, $\hat{\chi }_i(s)$, for $i=0,1,\cdots ,a-1$, are numerator coefficients terms and $\hat{\kappa }_i(s)$, for $i=0,1,\cdots ,a$ are denominator coefficients terms of ${Tf}_{a}(s)$. The HOS expressed in (5) is desired to be reduced to $b^{th}$ order. The LOM can be represented as:

6 $$\begin{aligned} {Tf}_{b}(s)= \frac{{\bar{\chi }_i(s)}}{{\bar{\kappa }_i(s)}} = \frac{{\bar{\chi }}_{0}+{\bar{\chi }}_{1}s+{\bar{\chi }}_{2}s^{2}+\ldots +{\bar{\chi }}_{b-1}s^{b-1}}{{\bar{\kappa }}_{0}+{\bar{\kappa }}_{1}s+{\bar{\kappa }}_{2}s^{2}+\ldots +{\bar{\kappa }}_{b}s^{b}} \end{aligned}$$

where, $\bar{\chi }_i(s)$ are the numerator terms in which *i* ranges from $i=0,1,\cdots ,b-1$ and $\bar{\kappa }_i(s)$ are denominator terms in which *i* ranges from $i=0,1,\cdots ,b$ of (6).

### Taylor and Laurent series expansion of HOS and LOM

The TS and LS are obtained by expansions around $s=0$ and $s=\infty$, respectively. The TS and LS of HOS are provided in (7) and (8). Similarly, for LOM TS and LS are presented in (9) and (10), respectively.

7 $$\begin{aligned} {Tf_{a}(s)}={\hat{\xi }}_0+{\hat{\xi }}_1s+{\hat{\xi }}_2s^2+\cdots +{\hat{\xi }}_a s^a+\cdots \end{aligned}$$

8 $$\begin{aligned} {Tf_{a}(s)}={\hat{\eta }}_1s^{-1}+{\hat{\eta }}_2s^{-2}+\cdots +{\hat{\eta }}_a s^{-a}+\cdots \end{aligned}$$

In (7), ${\hat{\xi }}_0$, ${\hat{\xi }}_1$, ${\hat{\xi }}_2$, $\cdots$, ${\hat{\xi }}_a$,$\cdots$ are TS coefficients while in (8), ${\hat{\eta }}_1$, ${\hat{\eta }}_2$, $\cdots$, ${\hat{\eta }}_a$,$\cdots$ are LS coefficients of HOS. The expansions of TS around $s = 0$ and LS around $s = \infty$ of (6) are represented as:

9 $$\begin{aligned} {Tf}_{b}(s)=\bar{\xi }_0+\bar{\xi }_1s+\bar{\xi }_2s^2+\cdots +\bar{\xi }_b s^b+\cdots \end{aligned}$$

10 $$\begin{aligned} {Tf}_{b}(s)=\bar{\eta }_1s^{-1}+\bar{\eta }_2s^{-2}+\cdots +\bar{\eta }_r s^{-b}+\cdots \end{aligned}$$

In (9), $\bar{\xi }_0$, $\bar{\xi }_1$, $\bar{\xi }_2$, $\cdots$, $\bar{\xi }_b$,$\cdots$ are TS coefficients, while in (10), $\bar{\eta }_1$, $\bar{\eta }_2$, $\cdots$, $\bar{\eta }_b$,$\cdots$ are LS coefficients of LOM.

### Formulation of fitness function and constraints of LOM

The formulation of fitness function is achieved by utilizing TS and LS of HOS and LOM. In fitness function, errors among TS and LS of HOS and LOM are supposed to be reduced. The comparable LOM can be obtained by knowing at least 2*b* terms of TS and LS. To develop fitness function, $b-1$ terms of TS and *b* terms of LS, among 2*b* terms of TS and LS, are considered. By utilizing $b-1$ terms and *b* terms of TS and LS, the objective function is formed in (11).

11 $$\begin{aligned} O_{f} = \sum _{p=1}^{b-1}\Bigg [{\bigg (1-\frac{{{\hat{\xi }_p}}}{{\bar{\xi }}_p}\bigg )}^2\Bigg ] + \sum _{g=1}^{b}\Bigg [{\bigg (1-\frac{{{\hat{\eta }_g}}}{\bar{\eta }_g}\bigg )}^2\Bigg ] \end{aligned}$$

**Fig. 2:**
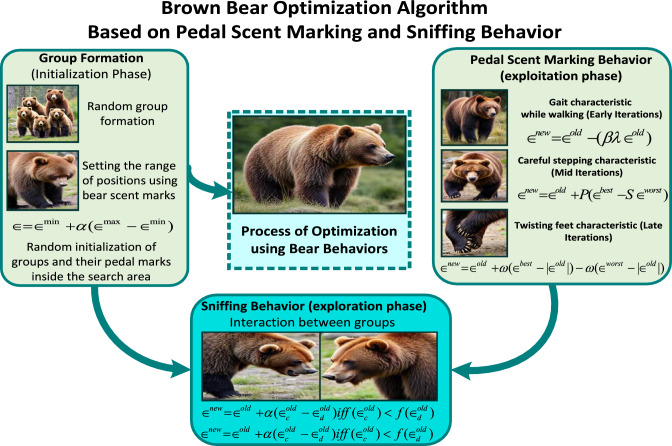
Behavior-inspired flow of the BBO Algorithm^24^.

In (11), the fitness function $O_{f}$ is the combination of three sub-objective functions. $\hat{\xi }_p$, $\bar{\xi }_p$, $\hat{\eta }_g$, and $\bar{\eta }_g$ are TS for HOS, TS for LOM, LS for HOS, and LS for LOM, respectively. For this paper, order diminution is done to obtain LOM of order 2. The choice of using a second-order model is primarily motivated by simplicity and control-oriented analysis for OMG applications, as the second-order model captures the dominant dynamic behavior of the HOS through its natural frequency and damping ratio. Therefore the value of $b=2$, (11) can be updated as follows,

12 $$\begin{aligned} O_{f} = {O_f}_1^{\xi }+{O_f}_1^{\eta }+{O_f}_2^{\eta } \end{aligned}$$

where ${O_f}_1^{\xi }$, ${O_f}_1^{\eta }$, and ${O_f}_2^{\eta }$ are sub-objective functions, and are mathematically represented in (13).

13 $$\begin{aligned} \begin{array}{cc} & {O_f}_1^{\xi }= \Big (1-\frac{{{\hat{\xi }_1}}}{{\bar{\xi }}_1}\Big )^2 \\ & {O_f}_1^{\eta }= \Big (1-\frac{{{\hat{\eta }_1}}}{{\bar{\eta }}_1}\Big )^2\\ & {O_f}_2^{\eta }= \Big (1-\frac{{{\hat{\eta }_2}}}{{\bar{\eta }}_2}\Big )^2 \end{array} \end{aligned}$$

The desired LOM is acquired by minimizing the fitness function given in (12) by satisfying the constraints of Hurwitz stability criterion and steady state matching. The Hurwitz stability criteria are used to obtain stable LOM and steady state matching ensures the zero steady state error. To ensures the zero steady state error the first TS expansion of both HOS and LOM presented in (8) and (10), respectively, are matched. This matching is shown in (14).

14 $$\begin{aligned} {\hat{\xi }}_0=\bar{\xi _0} \end{aligned}$$

Further, the stability of LOM (6) can be satisfied by applying Hurwitz stability criterion, i.e.,

15 $$\begin{aligned} \bar{\kappa }_i(s) \text { of }(6) \text { should satisfy Hurwitz criterion.} \end{aligned}$$

Thus, the construction of resultant fitness function is acquired in (12). The minimization of resultant fitness function is attained by exploiting the BBO algorithm. The description of BBO algorithm is presented in section "Brown Bear Optimization (BBO) Algorithm" (Fig. 2).

## Brown Bear Optimization (BBO) algorithm

Brown bears are among the largest territorial carnivorous animals, primarily found in Northern Eurasia and North America. The BBO algorithm was proposed by observing the communication behavior of brown bears. The well-known communication methods exhibited by brown bears include pedal scent marking and sniffing behavior^24,25^. This algorithm has been applied to power transmission systems, exploration phases for solving complex optimization problems, and economic dispatch problems. The adoption of this algorithm in this paper is motivated by its simple structure, suitability for multimodal error minimization problems, and the small number of control parameters, which reduces tuning effort. Its balanced exploration–exploitation mechanism enables effective global search and stable fine-tuning, making it particularly suitable for TS and LS-based model reduction under stability criteria. The mathematical representation of BBO algorithm reflects the natural behavioral characteristics of brown bears^26^.

Initially, a groups of brown bears are formed, where random solutions are generated. The pedal scent marks made by each bear are treated as decision variables within the solution sets. A population of brown bears is initialized considering the pedal scent-marking behavior within a defined territory, with a population size denoted by (*Y*) and the number of decision factors by (*Z*). The mathematical representation of random initialization of groups of brown bears within a territory is expressed in (16).

16 $$\begin{aligned} \epsilon _{e,f}=\epsilon ^{min}_{e,f}+\alpha (\epsilon ^{max}_{e,f}-\epsilon ^{min}_{e,f}) \end{aligned}$$

In (16), $\epsilon _{e,f}$ represents the $f^{th}$ pedal mark of the $e^{th}$ dimension, where $e = 1,2,\cdots ,Y$ and $f = 1,2,\cdots ,Z$. The terms $\epsilon ^{\min }_{e,f}$ and $\epsilon ^{\max }_{e,f}$ denote the minimum and maximum values of the pedal marks, respectively. The random variable $\alpha$ lies between 0 and 1. For the brown bear population, the complete solution set $\epsilon$ is represented as follows:

17 $$\begin{aligned} \epsilon =\begin{bmatrix} \epsilon _{1,1} & \epsilon _{1,2} & \cdots & \epsilon _{1,Z}\\ \epsilon _{2,1} & \epsilon _{2,2} & \cdots & \epsilon _{2,Z}\\ \vdots & \vdots & \ddots & \vdots \\ \epsilon _{Y,1} & \epsilon _{Y,2} & \cdots & \epsilon _{Y,Z}\\ \end{bmatrix} \end{aligned}$$

- **Phase 1: Pedal Scent Marking:** This phase represents the most characteristic walking behavior of brown bears. The pedal scent-marking behavior is primarily observed in male brown bears. During this process, a brown bear exhibits three main characteristics:
  - Gait characteristic while walking,
  - Careful stepping characteristic, and
  - Twisting feet characteristic
  The detailed descriptions of these three characteristics are presented below.
  1. Gait characteristic while walking: This characteristic refers to the bear’s natural gait and walking behavior. For simplification, each group is considered to have a single male brown bear. The pedal marks formed by each male bear differ significantly from others. It is assumed that the marks formed due to the gait characteristic occur during the first one-third of the total number of iterations ($R_{iter}$), as represented mathematically in (18).
    18 $$\begin{aligned} \epsilon ^{new}_{e,f,g}=\epsilon ^{old}_{e,f,g}-(\beta _{g}\cdot \lambda _{e,f,g}\cdot \epsilon ^{old}_{e,f,g}) \end{aligned}$$
    Here, $\epsilon ^{new}_{e,f,g}$ represents the updated solution, and $\epsilon ^{old}_{e,f,g}$ is the previous solution of the $f^{th}$ pedal mark of the $e^{th}$ group for the $k^{th}$ iteration. The variable $\lambda$ lies between 0 and 1. The occurrence factor, denoted by $\beta _g$, is the ratio of the current iteration ($R_{cl}$) to the total number of iterations ($R_{iter}$), as defined in (19).
    19 $$\begin{aligned} \beta _{g}=\frac{R_{cl}}{R_{iter}} \end{aligned}$$
  2. Careful stepping characteristic: This characteristic modifies the solution from one-third span to two-third span of $R_{iter}$. The mathematical representation of this phase is given in (20).
    20 $$\begin{aligned} \epsilon ^{new}_{e,f,g}=\epsilon ^{old}_{e,f,g}+ P_{g}(\epsilon ^{best}_{f,g}-S_{g}.\epsilon ^{worst}_{f,g}) \end{aligned}$$
    Here, $\epsilon ^{best}_{f,g}$ and $\epsilon ^{worst}_{f,g}$ represent the best and worst pedal scent marks among all iterations, respectively. $P_g$ denotes the step factor in the $g^{th}$ iteration, and its value depends on the occurrence factor $\beta _g$ expressed in (21). The parameters $P_g$ and $S_g$ are defined in (21) and (22).
    21 $$\begin{aligned} P_{g}=l_{1,g}.\beta _{g} \end{aligned}$$
    In (21), $l_{1,g}$ is a random variable that ranges between 0 and 1 at $g^{th}$ iteration. $S_g$ is the step dimension at $g^{th}$ and is given by
    22 $$\begin{aligned} S_{g}=round(1+l_{2,g}) \end{aligned}$$
    where $l_{2,g}$ is stochastic number evenly spread in interval [0,1].
  3. Twisting feet characteristic: Another distinctive behavior of brown bears is the twisting of their feet while walking, which helps them avoid previous ground depressions. This characteristic dominates during the final one-third of $R_{iter}$. Considering the angular velocity of the feet-twisting movement of a male brown bear on its previous pedal marks, the angular velocity for the $g^{th}$ iteration is expressed in (23).
    23 $$\begin{aligned} \omega _{e,g}= 2\pi .\beta _{g}.\phi _{e,g} \end{aligned}$$
    Here, $\phi _{e,g}$ is a random number uniformly distributed between 0 and 1. Using this twisting characteristic, the updated solution is given by (24).
    24 $$\begin{aligned} \epsilon ^{new}_{e,f,g}=\epsilon ^{old}_{e,f,g}+ \omega _{e,g}(\epsilon ^{best}_{f,g}-|\epsilon ^{old}_{e,f,g}|)-\omega _{e,g}(\epsilon ^{worst}_{f,g}-|\epsilon ^{old}_{e,f,g}|) \end{aligned}$$
- **Phase 2: Sniffing behavior**: The sniffing behavior allows brown bears to identify and interact with one another through the scent of pedal marks. This behavior enhances their ability to exchange information within the population. The mathematical representation of this behavior is given in (25) and (26).
  25 $$\begin{aligned} \epsilon ^{new}_{d,f,g}=\epsilon ^{old}_{d,f,g}+ \alpha _{e,g}(\epsilon ^{old}_{d,f,g}-\epsilon ^{old}_{c,f,g}) \textit{if \ \ } f(\epsilon ^{old}_{d,g})<f(\epsilon ^{old}_{c,g}) \end{aligned}$$
  26 $$\begin{aligned} \epsilon ^{new}_{d,f,g}=\epsilon ^{old}_{d,f,g}+ \alpha _{e,g}(\epsilon ^{old}_{c,f,g}-\epsilon ^{old}_{d,f,g}) \textit{if \ \ } f(\epsilon ^{old}_{c,g})<f(\epsilon ^{old}_{d,g}) \end{aligned}$$
  In (25) and (26), $\epsilon ^{new}_{d,f,g}$ is the updated solution for the $d^{th}$ individual, while $\epsilon ^{old}_{d,f,g}$ and $\epsilon ^{old}_{c,f,g}$ denote the previous values of the $d^{th}$ and $c^{th}$ individuals, respectively, where $c\ne d$. The fitness functions $f(\epsilon ^{old}_{d,g})$ and $f(\epsilon ^{old}_{c,g})$ correspond to the fitness values of the respective individuals.

**Algorithm 1:**
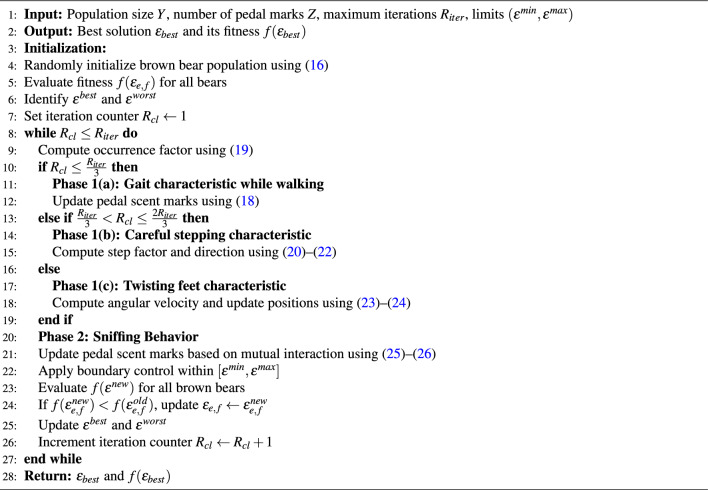
BBO algorithm

## Results and discussion

The transfer function of seventh-order OMG system is denoted by $Tf_{7}(s)$. The SOS is reduced to a SOM of OMG. The transfer function $Tf_{7}(s)$ is presented in (27).

27 $$\begin{aligned} Tf_{7}(s)= \frac{ 0.005642s^5+0.1461s^4+1.288s^3+4.536s^2+6.496s+3 }{ \begin{aligned}&0.0001221s^7+0.003822s^6+0.05656s^5+0.4938s^4+2.388s^3+5.61s^2+5.836s+2.014 \end{aligned} } \end{aligned}$$

The TS and LS is exploited in this study in (27), as TS captures low-frequency dynamics related with steady-state response, while LS shows high-frequency transient dynamics. The combined use of TS and LS ensures balanced preservation of both steady state and transient response. Unlike other conventional method which emphasize a limited frequency range, whereas the TS and LS approach provides balanced dynamic description over a wider frequency range. In addition, the TS and LS expansions directly operate on transfer function coefficients, allows direct implementation of minimum steady state error and stability constraints, and also avoids additional tuning parameters. The TS and LS expansions of (27) are presented in (28) and (29), respectively.

28 $$\begin{aligned} {Tf_7(s)}=1.489-1.088s+1.257s^2 -1.737s^3+\cdots \end{aligned}$$

29 $$\begin{aligned} {Tf_7(s)}=46.20s^{-2}-248.97s^{-3}-3063.06s^{-4}+\cdots \end{aligned}$$

This work proposes a SOM of OMG by substituting $y=2$ in (6). The proposed SOM is shown in (30).

**Fig. 3:**
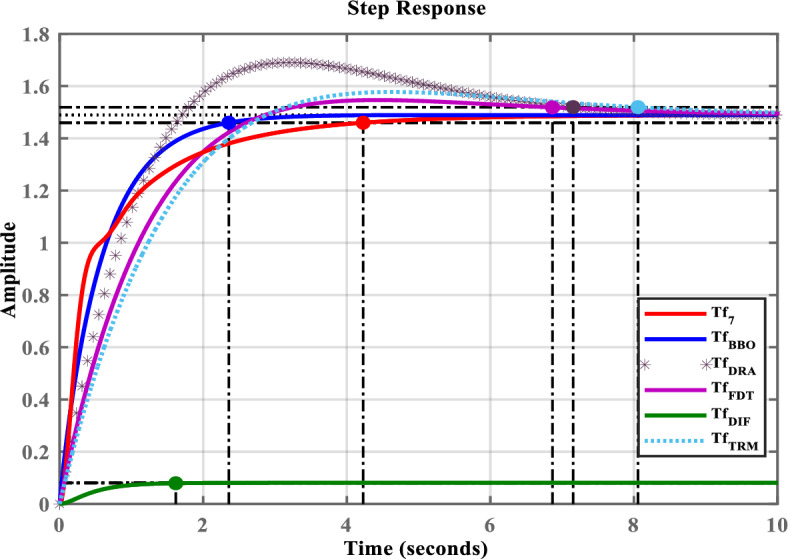
Step response of $Tf_{7}(s)$ and its LOMs.

**Fig. 4:**
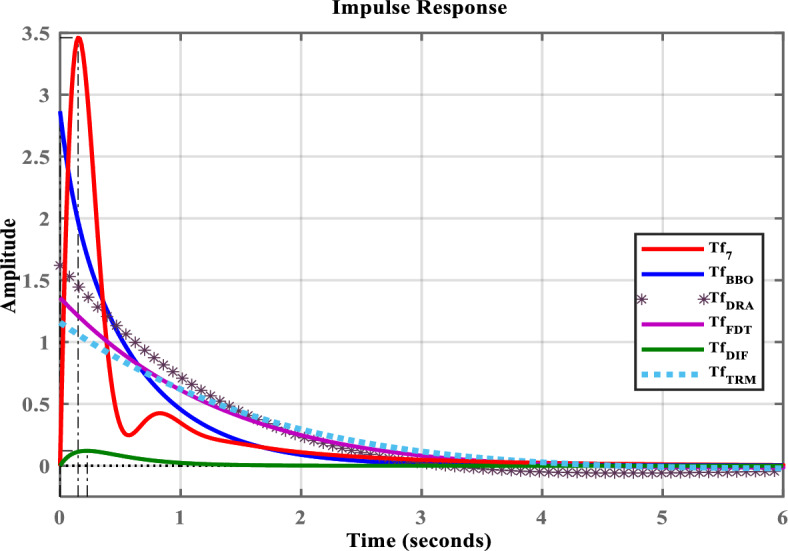
Impulse response of $Tf_{7}(s)$ and its LOMs.

30 $$\begin{aligned} Tf_2(s)=\frac{{\bar{\chi }}_{0}+{\bar{\chi }}_{1}s}{{\bar{\kappa }}_{0}+{\bar{\kappa }}_{1} s+{\bar{\kappa }}_{2}s^{2}} \end{aligned}$$

The expressions for TS and LS based on (9) and (10) of SOM are formed. These expansions are presented in (31) and (32), respectively.

31 $$\begin{aligned} Tf_2(s)= \frac{{\bar{\chi }}_{0}}{{\bar{\kappa }_{0}}}+ \ \frac{{\bar{\chi }}_{1}{\bar{\kappa }}_{0}-{\bar{\chi }}_{0}{\bar{\kappa }}_{1}}{{{\bar{\kappa }}_{0}}^2}s+ \frac{{\bar{\chi }}_{0}{{\bar{\kappa }}_{1}}^2-{\bar{\chi }}_{1}{\bar{\kappa }}_{0}{\bar{\kappa }}_{1}-{\bar{\chi }}_{0}{\bar{\kappa }}_{0}{\bar{\kappa }}_{2}}{{{\bar{\kappa }}_{0}}^3}s^2+ \cdots \end{aligned}$$

32 $$\begin{aligned} Tf_2(s) =\frac{{\bar{\chi }}_1}{{\bar{\kappa }}_2}s^{-1} + \ \frac{{\bar{\chi }}_0{\bar{\kappa }}_2-{\bar{\chi }}_1{\bar{\kappa }}_1}{{{\bar{\kappa }}_2}^2}s^{-2}+ \frac{{\bar{\chi }}_1{{\bar{\kappa }}_1}^{2}-{\bar{\chi }}_0 {\bar{\kappa }}_1{\bar{\kappa }}_2-{\bar{\chi }}_1{\bar{\kappa }}_0 {\bar{\kappa }}_2}{{{\bar{\kappa }}_2}^{3}}s^{-3}+ \cdots \end{aligned}$$

The overall fitness function is formed by putting the values of TS and LS from (28)-(32) in (12), as expressed in (33).

33 $$\begin{aligned} O_f={\Bigg (1-\Bigg (\frac{{\bar{\chi }}_1{\bar{\kappa }}_0-{\bar{\chi }}_0 {\bar{\kappa }}_1}{-1.088{{\bar{\kappa }}_{0}}^{2}}\Bigg )\Bigg )}^{2}+ {\Bigg (1-\Bigg (\frac{{\bar{\chi }}_0 {\bar{\kappa }}_2-{\bar{\chi }}_1{\bar{\kappa }}_1}{46.20{{\bar{\kappa }}_2}^{2}}\Bigg )\Bigg )}^{2}+ {\Bigg (1-\Bigg (\frac{{\bar{\chi }}_1{{\bar{\kappa }}_1}^{2}-{\bar{\chi }}_0 {\bar{\kappa }}_1{\bar{\kappa }}_2-{\bar{\chi }}_1{\bar{\kappa }}_0 {\bar{\kappa }}_2}{-248.97{{\bar{\kappa }}_2}^{3}}\Bigg )\Bigg )}^{2} \end{aligned}$$

In the presented formulation (33), each TS and LS based term is described as a normalized squared error between the original system and the desired LOM (30), that results in reduced coefficient sensitivity imbalance and uniform numerical scaling. Hence, in this study no weighting techniques or direct coefficient sensitivity analysis is incorporated. Instead of that equal weighting is used by assigning the value unity to all sub-objective functions to ensure unbiased contribution from both low and high-frequency dynamics.

**Table 2:** Comparative assessment of time domain specifications of $Tf_{7}(s)$ and its LOMs.

$Tf_{7}(s)$, its LOMs	Rise Time (s)	Transient Time (s)	Settling Time (s)	Overshoot	Peak	Peak Time (s)
$Tf_{7}(s)$, (27)	1.8555	4.2280	4.2280	0	1.4883	8.9622
$Tf_{BBO}(s)$, (35)	1.3213	2.3570	2.3570	0	1.4888	4.4566
$Tf_{DRA}(s)$, (36)	1.2579	7.1536	7.1536	13.4781	1.6903	3.1972
$Tf_{FDT}(s)$, (37)	1.8500	6.8655	6.8655	3.8864	1.5466	4.4578
$Tf_{DIF}(s)$, (38)	0.9010	1.6177	1.6177	0	0.0808	2.9039
$Tf_{TRM}(s)$, (39)	1.9798	8.0569	8.0569	5.9022	1.5775	4.6039

The BBO algorithm is implemented with population size 100, maximum iteration count is 100, and the step factors are the random variables value lies between [0, 1] to support a balanced exploration and exploitation mechanism. The decision variable bounds are in the range of [−10,10] that ensures stability and a stable search space. The algorithm convergence behavior is analyzed by observing the best fitness value across iterations, and convergence is achieved when the proposed model attains the minimum error between the SOS and the SOM. By utilizing the BBO algorithm, the error of fitness function presented in (33) is minimized. The optimization is performed under two conditions, namely Hurwitz stability and steady-state error. The steady-state matching and stability conditions are provided in (14) and (15), respectively. To solve (33), constraints (14) and (15) are modified and then the updated expressions are obtained in (34).

34 $$\begin{aligned} {\bar{\chi }_0=1.489\bar{\kappa }_0},\ {\bar{\kappa }_1>0, \ \ \bar{\kappa }_0 \bar{\kappa }_1>0} \end{aligned}$$

**Table 3:** Frequency-domain stability margin analysis of $Tf_{7}(s)$ and its LOMs.

$Tf_{7}(s)$, its LOMs	GM (dB)	PM (deg)	$\boldsymbol{\omega _{gc}} (rad/sec)$	$\boldsymbol{\omega _{pc}} (rad/sec)$	DM (s)	Stable
$Tf_{7}(s)$, (27)	$\infty$	146.51	$\infty$	1.6371	1.5620	Yes
$Tf_{BBO}(s)$, (35)	$\infty$	133.71	$\infty$	1.8388	1.2691	Yes
$Tf_{DRA}(s)$, (36)	$\infty$	117.58	$\infty$	1.5365	1.3356	Yes
$Tf_{FDT}(s)$, (37)	$\infty$	125.01	$\infty$	1.1644	1.8738	Yes
$Tf_{DIF}(s)$, (38)	$\infty$	$\infty$	$\infty$	$\infty$	$\infty$	Yes
$Tf_{TRM}(s)$, (39)	$\infty$	121.81	$\infty$	1.0568	2.0117	Yes

**Table 4:** Quantitative frequency-domain performance comparison.

$Tf_{7}(s)$, its LOMs	Bandwidth (rad/s)	Bandwidth error (%)	Peak deviation (dB)
$Tf_{7}(s)$, (27)	13.3819	–	–
$Tf_{BBO}(s)$, (35)	4.1102	69.29	15.77
$Tf_{DRA}(s)$, (36)	3.0717	77.05	10.82
$Tf_{FDT}(s)$, (37)	2.4320	81.83	12.29
$Tf_{DIF}(s)$, (38)	2.6445	80.24	33.38
$Tf_{TRM}(s)$, (39)	2.1450	83.97	13.66

**Table 5:** Comparison of dominant damping characteristics between $Tf_{7}(s)$ and its LOMs.

$Tf_{7}(s)$, its LOMs	Dominant pole type	Damping ratio	Frequency (rad/s)
$Tf_{7}(s)$, (27)	Real poles	1.000	1.84
$Tf_{BBO}(s)$, (35)	Real poles	1.000	1.64
$Tf_{DRA}(s)$, (36)	Complex pair	0.833	0.71
$Tf_{FDT}(s)$, (37)	Complex pair	0.940	0.65
$Tf_{DIF}(s)$, (38)	Real poles	1.000	2.73
$Tf_{TRM}(s)$, (39)	Complex pair	0.868	0.60

**Table 6:** Comparative assessment of performance error criteria of LOMs concerning $Tf_{7}(s)$.

LOMs	ISE	ITSE	IT$^2$SE	IAE	ITAE	IT$^2$AE
$Tf_{BBO}(s)$, (35)	0.02548	0.06535	0.203	0.3322	1.098	4.567
$Tf_{DRA}(s)$, (36)	0.2442	0.9066	3.936	1.188	4.982	24.95
$Tf_{FDT}(s)$, (37)	0.1381	0.3083	1.011	0.7454	2.732	14.01
$Tf_{DIF}(s)$, (38)	15.47	92.61	643	11.63	67.46	462.3
$Tf_{TRM}(s)$, (39)	0.2096	0.515	1.904	0.9802	3.814	20.62

A SOM of OMG is obtained by implementing BBO algorithm subject to constraints given in (34). The model is presented in (35).

35 $$\begin{aligned} Tf_{BBO}(s)=\frac{0.6778s+5.141}{0.2365s^2+2.489s+3.451} \end{aligned}$$

The proposed model in (35) is compared with the models presented in (36), (37), (38), and (39). These models are derived by applying classical MOD techniques to the seventh-order transfer function $(Tf_{7}(s))$ given in (27). The model $Tf_{DRA}(s)$ in (36) is derived by applying direct truncation to the numerator and the Routh approximation method^27,28^ to the denominator of $Tf_{7}(s)$. The factor division technique^29^ is applied to the SOS in $Tf_{7}(s)$ of the OMG, resulting in the model $Tf_{FDT}(s)$ shown in (37). By applying the differentiation technique^30^ to $Tf_{7}(s)$, the corresponding reduced model $Tf_{DIF}(s)$ is obtained as given in (38). The truncation reduction technique^31^ is applied to the OMG system in $Tf_{7}(s)$, and the resulting model $Tf_{TRM}(s)$ is presented in (39).

36 $$\begin{aligned} Tf_{DRA}(s)=\frac{6.496s+3}{4.0118s^2+4.7383s+2.014} \end{aligned}$$

37 $$\begin{aligned} Tf_{FDT}(s)=\frac{1.3575s+0.626}{s^2+1.219s+0.4205} \end{aligned}$$

38 $$\begin{aligned} Tf_{DIF}(s) = \frac{54.432}{35.553s^{2} + 343.872s + 673.2} \end{aligned}$$

39 $$\begin{aligned} Tf_{TRM}(s)=\frac{6.496s+3}{5.61s^2+5.836s+2.014} \end{aligned}$$

**Fig. 5:**
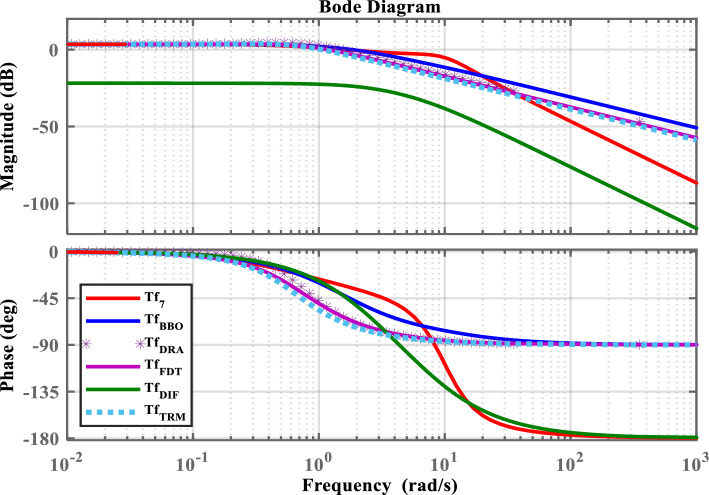
Bode response of $Tf_{7}(s)$ and its LOMs.

**Fig. 6:**
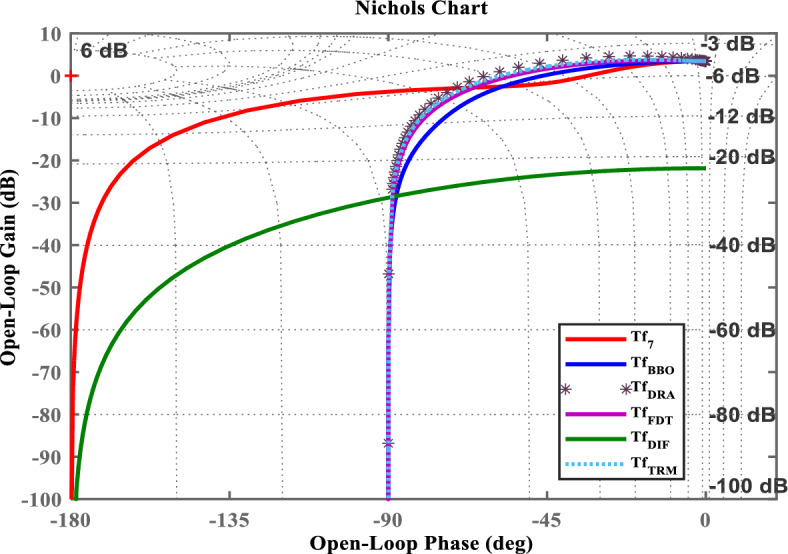
Nichols chart of $Tf_{7}(s)$ and its LOMs.

**Fig. 7:**
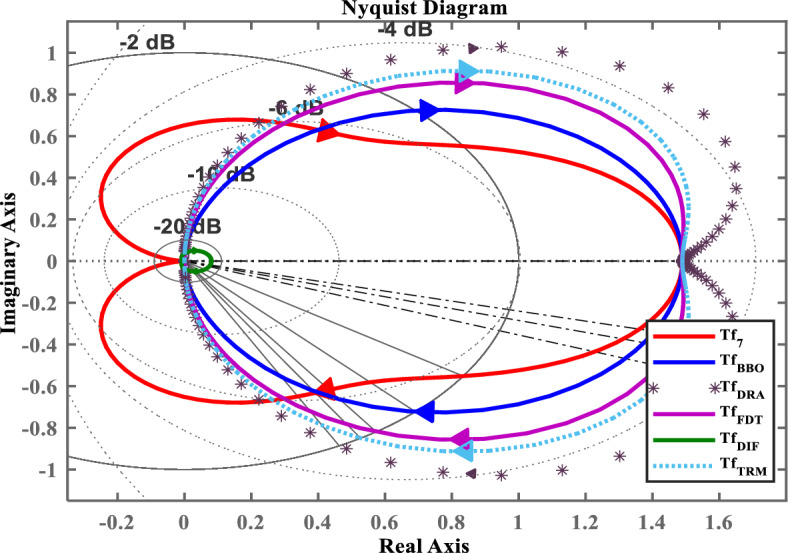
Nyquist response of $Tf_{7}(s)$ and its LOMs.

**Fig. 8:**
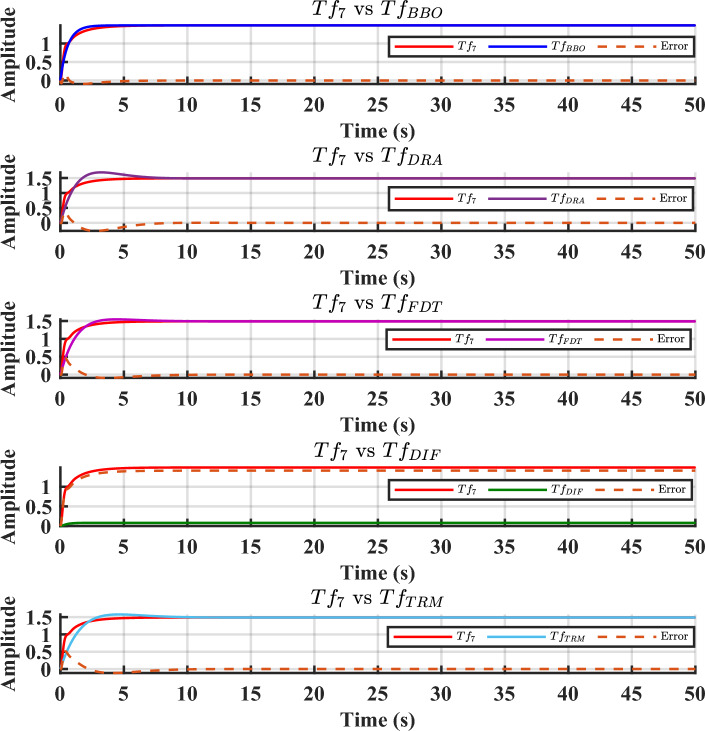
Comparison of step response and error between $T_{f7}(s)$ and its LOMs.

**Fig. 9:**
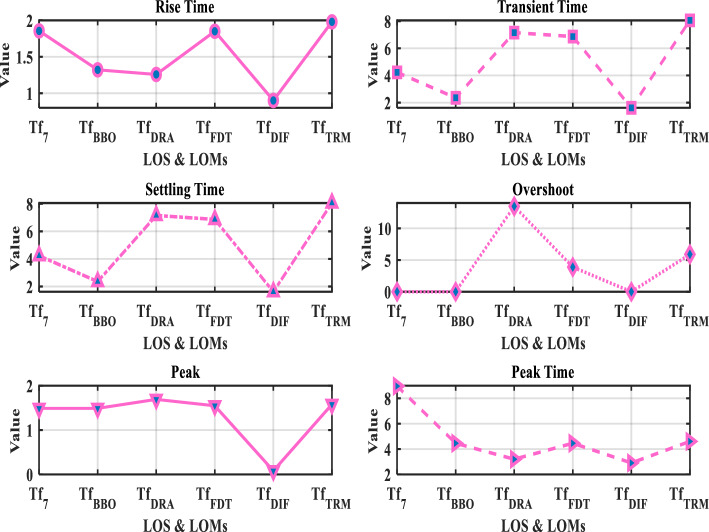
Comparative assessment of time domain specifications of $Tf_{7}(s)$ and its LOMs.

**Fig. 10:**
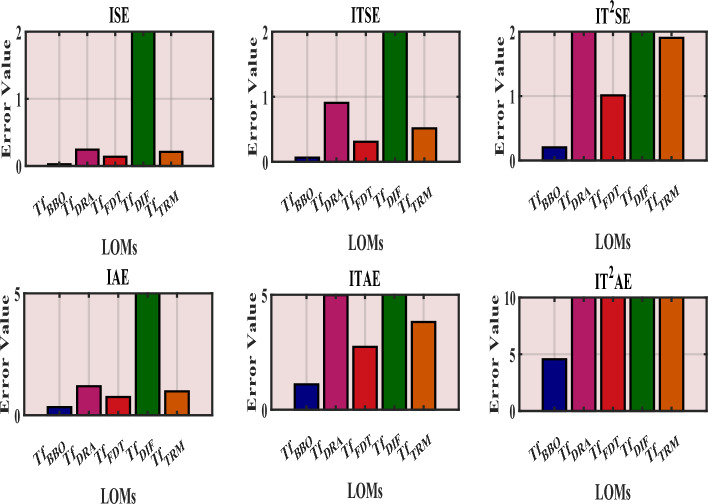
Comparative assessment of error indices of LOMs concerning $Tf_{7}(s)$.

To demonstrate the efficacy of the proposed approach, the obtained model in (35) is compared with the obtained models shown in (36), (37), (38), and (39). For graphical comparison, the time-domain analysis that includes step and impulse responses is illustrated in Figs. 3,4, respectively. As observed from Figs. 3,4, the response of the proposed model (35) closely matches the original system response (27), compared to the other LOMs shown in (36)-(39). Furthermore, to provide a clearer understanding the frequency-domain characteristics, the Bode, Nichols, and Nyquist plots of the system and its LOMs are presented in Figs. 2,3,4,5,6,7, respectively. A comparative evaluation is also provided in tabular form, listing the time-domain specifications (TDSs), frequency-domain specifications (FDSs), and performance error criteria (PECs) of the system and its LOMs (Fig. 8).

Table 2 presents the TDSs, including rise time, transient time, settling time, overshoot, peak value, and peak time of $Tf_{7}(s)$ and its LOMs. It is clearly observed that the TDSs of the proposed model (35) closely resemble the original system (27) when compared to other LOMs obtained through (36)–(39). The peak value of the original system (27) is 1.4883, which is exactly equal to that of the proposed model (35). Similarly, the settling time of (27) is 4.2280, which is approximately comparable to that of (35) 2.3570. Both models exhibit zero overshoot. To clearly illustrate this comparison, the corresponding plot is shown in Fig. 9.

Tables 3,4,5 present the quantitative metrics of the frequency-domain specifications (FDSs). Table 3 reports the gain margin (GM), phase margin (PM), crossover frequencies $\boldsymbol{\omega _{gc}}$, $\boldsymbol{\omega _{pc}}$, and delay margin (DM), thereby providing a comprehensive frequency-domain stability margin analysis of $Tf_{7}(s)$ and its LOMs. Table 4 presents a quantitative frequency-domain performance comparison, including bandwidth, bandwidth error, and peak deviation for $Tf_{7}(s)$ and its LOMs. By analyzing the Table 3 and 4, the BBO-based LOM (35) maintains a closely high phase margin (133.71$^\circ$), nominal bandwidth error (69.29%), and moderate peak deviation (15.77 dB), that shows a balance between frequency-response matching and stability robustness with original system (27). Table 5 compares the dominant damping characteristics of $Tf_{7}(s)$ and its LOMs, with dominant pole type, damping ratio, and dominant frequency systematically tabulated. Particularly, the analysis of the data presented in Table 5 shows that $Tf_{7}(s)$ and its BBO-based LOM $Tf_{BBO}(s)$ exhibit real dominant poles with a damping ratio of 1.0 and closely matching dominant frequencies of 1.84*rad*/*s* and 1.64*rad*/*s*, respectively.

Table 6 summarizes the PECs between $Tf_{7}(s)$ and its LOMs in terms of the integral square error (ISE), integral time square error (ITSE), integral time-squared square error (IT$^{2}$SE), integral absolute error (IAE), integral time absolute error (ITAE), and integral time-squared absolute error (IT$^{2}$AE). From Table 6, it can be observed that the PECs values of the proposed model (35) are consistently lower than those of other models derived in (36)–(39). Hence, the proposed LOM (35) demonstrates superior performance in terms of accuracy and dynamic behavior. The graphical comparison of PECs is illustrated in Fig. 10. From Table 6 and Fig. 10, it is evident that $Tf_{BBO}(s)$ exhibits the lowest errors, with ISE = 0.02548, ITSE = 0.06535, IT$^{2}$SE = 0.203, IAE = 0.3322, ITAE = 1.098, and IT$^{2}$AE = 4.567, confirming its minimal deviation from the original system.

Considering all the responses presented in Fig(s). 3–7, along with the tabulated data in Tables 2-6 and their graphical representations in Fig(s). 8-10, it can be conclusively stated that among all the LOMs, the proposed model (35) most closely replicates the dynamic and steady-state characteristics of the original system (27) while achieving the minimum error values.

## Conclusion

The presented work addresses the lower-order (LO) modeling of a higher-order off-grid microgrid (OMG) system using optimization-based approaches. In this study, higher-order system (HOS) are reduced to lower-order model (LOM) while preserving their essential dynamic characteristics. The Taylor and Laurent series expansions are employed to construct the fitness functions. The resultant fitness functions are then minimized using the brown bear optimization (BBO) algorithm, subject to the Hurwitz stability criterion and the condition of zero steady-state error. This ensures both the stability and accuracy of the resulting LOM. The effectiveness of the proposed approach is validated through comparative analysis with LOMs obtained using existing reduction techniques. Step, impulse, Bode, Nichols, and Nyquist plots are presented to demonstrate the effectiveness of the proposed approach. Additionally, time-domain parameters, frequency domain parameters and error criteria are tabulated and graphically presented to further validate the results.

The present work proposes a second-order BBO-based reduced model. Although reducing the system order to two may lead to the loss of certain dynamic characteristics of the original higher-order system, the proposed reduced model is preferable for system-level analysis and preliminary control design, as it effectively resembles the dominant dynamics. However, some internal interactions under extreme operating conditions may not be fully represented. To address this limitation, future work will focus on developing LOMs that can capture the essential dynamics of EV-integrated OMG more accurately. In addition, future work may explore systematic weight-assessment strategies, such as rank exponent and proximity index techniques, can be explored. Furthermore, the applicability of the proposed methodology can be extended by employing different optimization techniques to minimize error more effectively.

## Data Availability

The datasets used and/or analyzed during the current study are available from the corresponding author upon reasonable request.
